# Whether joint leisure time physical activity and dietary quality alleviates metabolic syndrome and its components: evidence from the National Health and Nutrition Examination Survey (2007–2018)

**DOI:** 10.1371/journal.pone.0322608

**Published:** 2025-05-06

**Authors:** Jingyi Xie, Bin Wang

**Affiliations:** School of Physical Education, Central China Normal University, Wuhan, China; University of Virginia, UNITED STATES OF AMERICA

## Abstract

**Objectives:**

The incidence of metabolic syndrome (MetS) is increasing, which is one of the major threats to human health. Whether joint leisure time physical activity (LTPA) and dietary quality (DQ) can reduce the risk of developing MetS and its components is worth exploring. Therefore, this study aimed to investigate the individual and combined effects of LTPA and DQ on MetS and its components.

**Methods:**

Data were extracted from the National Health and Nutrition Examination Survey from 2007 to 2018. LTPA was classified as inactive, insufficiently active (IA), weekend warrior (WW), and regular active (RA); DQ was categorized as high dietary quality (HDQ) and low dietary quality (LDQ). Afterwards, population characteristics of MetS were analyzed. Then, univariate and multivariate logistic regression were used to analyze individual and combined effects of LTPA and DQ on MetS and its components. Subgroup analysis and restricted cubic spline (RCS) were used to examine the robustness and non-linearity.

**Results:**

This study included 31,482 adults aged 20 or older. Results showed that RA, HDQ and RA&HDQ were significantly negatively correlated with MetS and its components. IA&HDQ was significantly and negatively correlated with MetS, waist circumference and high-density lipoprotein cholesterol. WW&HDQ was not significantly associated with MetS, but was significantly negatively linked to fasting glucose and blood pressure. No significant interaction effect was observed in subgroup analysis. RCS analysis revealed a significant non-linear negative correlation between LTPA and MetS.

**Conclusions:**

This research indicates that RA and HDQ alone or in combination are associated with a lower risk of developing MetS and its components and could serve as effective preventive and therapeutic strategies against MetS and its associated risk factors. The decline in MetS risk become even more significant after exercise for 500 min/week. Similar trends are observed among fasting glucose, blood pressure, and triglyceride levels.

## Introduction

Metabolic syndrome (MetS), characterized by abdominal obesity, hypertension, abnormal blood sugar levels, high-density lipoprotein cholesterol, and triglyceride, refers to the presence of multiple metabolic abnormalities in the human body [1]. Data indicate that the global prevalence of MetS is approximately one-fourth of the total population, with over one billion people affected worldwide [2]. MetS is associated with a 2.5-fold increased risk of cardiovascular mortality, a 5-fold increased risk of diabetes, a 2-fold increased risk of coronary and cerebrovascular disease, and a 1.5-fold increased risk of all-cause mortality [3]. MetS has become a serious public health problem, making early detection and intervention crucial topics in the field. Leisure time physical activity (LTPA) and diet quality (DQ) are key lifestyle factors that significantly influence the occurrence and development of MetS. Several studies have reported the importance of DQ and LTPA in protecting against MetS [4,5].

LTPA is one of the most widely used and cost-effective methods to reduce the risk of chronic diseases [6]. The American College of Sports Medicine and World Health Organization recommend that people should perform at least 75 min of vigorous-intensity physical activity (VPA) or 150 min of moderate-intensity physical activity (MPA) per week [7,8]. However, variations in people’s work and lifestyle lead to considerable differences in the duration and frequency of participating in LTPA. Exercise types are categorized into regular exercise, weekend exercise, insufficient exercise, no exercise, and others categories based on exercise frequency and duration [9–12]. The literature indicates that distinct exercise patterns may elicit vastly different effects. Some studies suggest that engaging in LTPA more than three times a week has significant benefits for reducing chronic disease conditions; whereas others indicate that even 1–2 sessions of exercise per week can contribute to reducing health risks, including improving cardiovascular disease [11], alleviating depressive symptoms [10], and so on. However, there are also studies arguing that the effectiveness of weekend-only exercise may be limited due to its relatively low frequency [9]. As social competition intensifies and the pace of life accelerates, individuals are increasingly inclined to exercise on weekend [12,13]. Nevertheless, the relationship between different exercise patterns and MetS is still unknown.

DQ also plays an important role in the management of MetS [14]. High dietary quality (HDQ) is thought to reduce the risk of MetS by helping individuals control their total calorie intake and maintain lower lipid levels by increasing their consumption of high fiber, high protein, and low carbohydrate food [15]. A cross-sectional study found a significant negative association between DQ and MetS in Iranian women [16], which was also supported by [17]. However, one study found no significant differences in the DQ between individuals with and without MetS [18]. Therefore, this study was conducted to further explore the relationship between DQ and MetS.

In addition to the independent effect of LTPA and DQ on MetS, the combination of LTPA and DQ may yield more significant benefits than either intervention alone [19,20]. Therefore, it is worth exploring whether the combination of LTPA and DQ can reduce the risk of developing MetS and its components.

In summary, using data from the National Health and Nutrition Examination Survey (NHANES) from 2007 to 2018, this study aimed to investigate the baseline characteristics of MetS in the U.S. population and to examine the individual effects of LTPA and DQ on MetS and its components. Furthermore, the effects of different combinations of LTPA and DQ on MetS and its components were also analyzed.

## Methods

### Study population

**Fig 1:**
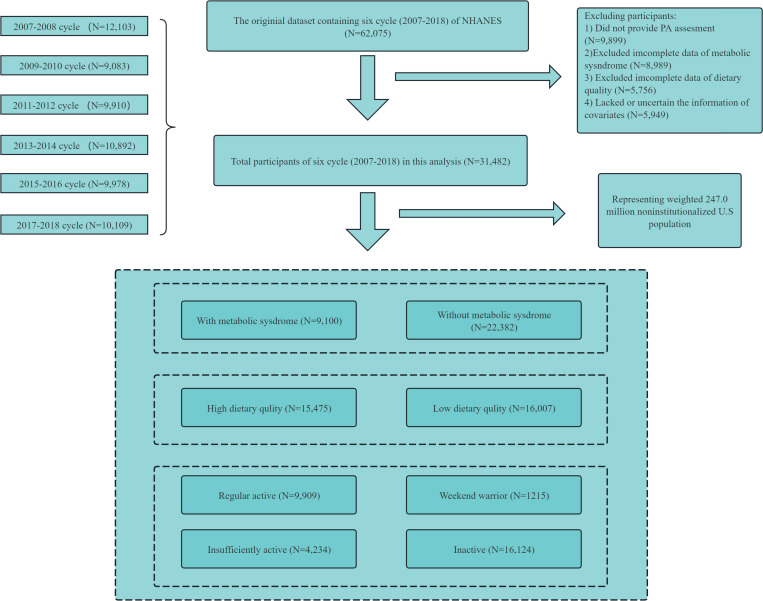
A flowchart showing the selection of study participants.

The NHANES is a cross-sectional survey conducted by the United States Centers for Disease Control and Prevention (https://www.cdc.gov/nchs/nhanes/index.htm). The protocol of the NHANES study was approved by the Ethics Review Committee of the National Center for Health Statistics (NCHS) [21]. This study used data from six NHANES cycles from 2007 to 2018. In total, 31,482 NHANES participants aged ≥20 years were included after excluding those with incomplete LTPA, DQ, MetS, and covariate data (**Fig 1**).

### Assessment of LTPA

The Global Physical Activity Questionnaire was used to collect data on the frequency and duration of VPA/MPA during participants’ leisure time in the past 7 days [22]. LTPA was assessed according to whether participants engaged in VPA/MPA exercise, the frequency of these activities, and their duration over a 7-day period. Total LTPA, also known as MVPA, was obtained by adding twice the number of minutes of VPA plus the number of minutes of MPA; 1 min of VPA is equivalent to 2 min of MPA [8]. Total LTPA was then categorized into four patterns of exercise: inactive (no MVPA), insufficiently active (IA; < 150 min), WW (≥150 min MVPA duration per week in 1 or 2 sessions), and regular active (RA; ≥ 150 min MVPA per week in 3 or more sessions).

### Assessment of DQ

Healthy Eating Index-2015 (HEI-2015) was used to assess DQ. HEI-2015 scores ranging from 0 to 100 were derived from two 24-h dietary recalls conducted 3–10 days apart [23,24], where a higher score indicated a higher overall DQ [25,26]. An average score at or above the 60th percentile of the HEI was considered to indicate HDQ; otherwise, it was considered to be low diet quality (LDQ).

### Definitions of lifestyle groups for LTPA and DQ

According to the included variables, eight lifestyle categories were created: [1] inactive&LDQ, [2] IA&LDQ, [3] WW&LDQ, [4] RA&LDQ, [5] inactive&HDQ, [6] IA&HDQ, [7] WW&HDQ, and [8] RA&HDQ. For subsequent logistic regression models, inactive&LDQ was set as the reference group.

### Assessment of MetS

MetS was defined according to the National Cholesterol Education Program [27,28]. The criteria are as follows: [1] triglyceride ≥ 1.69 mmol/L (150 mg/dL); [2] high-density lipoprotein cholesterol: male high-density lipoprotein cholesterol < 1.03 mmol/L (40 mg/dL) and female high-density lipoprotein cholesterol < 1.29 mmol/L (50 mg/dL); [3] fasting glucose ≥ 6.1 mmol/L (1 mmol/L 110 mg/dL); [4] waist circumference: male waist circumference > 102 cm and female waist circumference > 88 cm; and [5] blood pressure ≥ 130/85 mmHg.

### Assessment of covariates

The covariates identified in this study included sex (male or female), age, race or ethnicity [(non-Hispanic white, non-Hispanic black, Mexican American, other race (including multiracial and other Hispanics)], education level (less than a high school diploma, high school graduate, or higher than a high school graduate), marital status (widowed or divorced, never married, married, or living with a partner), and poverty to income ratio (PIR) as an estimate of socioeconomic status. Self-reported lifestyle factors were also considered, including alcohol consumption, body Mass Index (BMI), total energy intake, smoking status (never, mild, moderate, or heavy), presence of heart disease (yes or no), presence of arthritis (yes or no), duration of sedentary behavior, and sleep duration.

### Statistical analyses

According to the analysis guidelines published by the National Center for Health Statistics (NCHS), the National Health and Nutrition Examination Survey (NHANES) utilizes complex, multi-stage probabilistic sampling designs that account for stratification and primary sampling units [29]. These designs guarantee representativeness of the sample within the U.S. population. The appropriate weight (WTDR2D) was selected for analysis. Moreover, to compute the weighted population representation, a 2-year sample weight was divided by six, reflecting the six cycles encompassed in the study.

Continuous variables were described as the mean ± standard deviation (M ± SD), while categorical variables were presented as count and weighted percentage (%). Adjusted odds ratios (AOR) with 95% confidence intervals (CI) were used to assess the different variables. Univariate and multivariate logistic regression models were used to adjust for covariates. Model 1 was an unadjusted model; Model 2 was adjusted for survey cycle, sex, age, race, education, PIR, marital status; and Model 3 was adjusted for survey cycle, sex, age, race, education, PIR, marital status, BMI, alcohol use, smoking, sleep duration, sedentary duration, total energy intake, arthritis and heart attack. Subgroup analysis and restricted cubic spline (RCS) were performed to examine the robustness and non-linearity of the associations between different variables. All statistical analyses were performed using R-4.1.0. All statistical tests were two-sided, and a *P-*value of < 0.05 was considered statistically significant.

## Results

### General characteristics

As presented in **Table 1**, this analysis included 31,482 individuals aged 20–80 years across six NHANES survey cycles, representing approximately 247.0 million U.S. adults. The mean age of participants was 42.46 ± 19.67 years; 15,299 were males (48.5%), and 16,183 were females (51.5%). Non-Hispanic Whites constituted the highest proportion of all participants, accounting for 65.9% (N = 12,905). Additionally, individuals who completed high school education represented the majority of the total population, totaling 13,696 (53.9%). Among all participants, the number of those with a PIR exceeding 3.5 was the highest, amounting to 8,995 (41.1%). The number of individuals who reported having moderate alcohol consumption habits was also prominent, reaching 19,989 (59.5%). Meanwhile, the vast majority of participants (20,598, 62.9%) stated that they did not smoke. In terms of the overall population, the average BMI was 28.23 ± 7.12, the average sleep duration was 6.92 ± 1.05 hours, and the sedentary time was 381.99 ± 194.79 minutes. Furthermore, arthritis and heart attack were the most prevalent conditions among participants, affecting 6,964 (22.3%) and 1,070 individuals (2.8%) respectively. In the assessment of the HEI-2015, participants scored an average of 52.84 ± 13.33, and they consumed an average of 2079 kilocalories per day. Regarding physical activity, the mean duration was 255.75 ± 453.76 minutes. Notably, the inactive group comprised the largest proportion of the total population, totaling 16,124 (45.1%).

**Table 1:** Baseline characteristics of participants stratified by MetS.

Characteristics	Without MetS (N = 22382)	With MetS (N = 9100)	Overall (N = 31482)	P value
Age (SD)	38.27 (19.24)	53.06 (16.50)	42.46 (19.67)	**<0.001**
Sex (%)				**<0.001**
Men	10159 (65.8%)	5140 (34.2%)	15299 (48.5%)	
Women	12223 (77.1%)	3960 (22.9%)	16183 (51.5%)	
Race (%)				
Non-Hispanic White	8728 (70.5%)	4177 (29.5%)	12905 (65.9%)	**<0.001**
Non-Hispanic Black	5294 (77.9%)	1647 (22.1%)	6941 (11.4%)	**<0.001**
Mexican American	3428 (69.9%)	1542 (30.1%)	4970 (9.3%)	**0.042**
Other Hispanic	2150 (70.7%)	964 (29.3%)	3114 (5.6%)	0.429
Other races	2782 (75.0%)	770 (25.0%)	3552 (7.8%)	**0.020**
Education level (%)				
<High school	9160 (79.2%)	2615 (20.8%)	11775 (25.7%)	**<0.001**
High school	3772 (63.4%)	2239 (36.6%)	6011 (20.4%)	**<0.001**
> High school	9450 (71.1%)	4246 (28.9%)	13696 (53.9%)	0.241
PIR (%)				
<1.3	7540 (71.4%)	3078 (28.6%)	10618 (23.5%)	0.806
1.3-3.5	8277 (70.6%)	3592 (29.4%)	11869 (35.4%)	0.071
>3.5	6565 (72.6%)	2430 (27.4%)	8995 (41.1%)	0.072
Marital status (%)				
Married or living with partner	8997 (65.5%)	5494 (34.5%)	14491 (53.0%)	**<0.001**
Widowed, divorced or separated	10329 (88.1%)	1225 (12.0%)	11554 (31.5%)	**<0.001**
Never married	3056 (59.0%)	2381 (41.0%)	5437 (15.4%)	**<0.001**
Alcohol use (%)				
Never	2171 (66.2%)	1210 (33.7%)	3381 (9.1%)	**<0.001**
Mild	14557 (71.9%)	5432 (28.1%)	19989 (59.5%)	0.503
Moderate	2578 (74.0%)	1025 (26.1%)	3603 (14.3%)	**0.032**
Heavy	3076 (71.7%)	1433 (28.3%)	4509 (17.1%)	0.926
Smoke (%)				**<0.001**
No	15997 (76.8%)	4601 (23.2%)	20598 (62.9%)	
Yes	6385 (62.8%)	4499 (37.2%)	10884 (37.1%)	
BMI (SD)	26.52 (6.55)	32.54 (6.66)	28.23 (7.12)	**<0.001**
Sleep duration, hours (SD)	6.93 (1.02)	6.90 (1.12)	6.92 (1.05)	0.176
Sedentary duration, minutes (SD)	378.97 (192.11)	389.62 (201.20)	381.99 (194.79)	**0.002**
HEI-2015 (SD)	53.00 (13.48)	52.42 (12.96)	52.84 (13.33)	**0.015**
Total energy intake, kcal (SD)	2061.26 (794.29)	2125.71 (824.05)	2079.55 (803.36)	**<0.001**
Arthritis (%)				**<0.001**
No	18842 (76.7%)	5676 (23.3%)	24518 (77.7%)	
Yes	3540 (53.8%)	3424 (46.2%)	6964 (22.3%)	
Heart attack (%)				**<0.001**
No	22000 (72.6%)	8412 (27.4%)	30412 (97.2%)	
Yes	382 (35.8%)	688 (64.2%)	1070 (2.8%)	
LTPA duration, minutes (SD)	296.34 (490.91)	153.33(320.46)	255.75 (453.76)	**<0.001**
LTPA pattern (%)				
Inactive	10682 (65.3%)	5442 (34.7%)	16124 (45.1%)	**<0.001**
Insufficiently active	2913 (70.3%)	1321 (29.7%)	4234 (15.2%)	0.226
WW	912 (73.6%)	303 (26.5%)	1215 (4.2%)	0.389
Regularly active	7875 (79.9%)	2034 (20.1%)	9909 (35.5%)	**<0.001**

From Table 1, the rate of MetS among males (34.2%) is higher than among females (22.9%). Mexican American exhibit a greater prevalence of MetS compared to other racial groups. The rate of MetS among individuals with a high school education is higher than those with other educational backgrounds. Moreover, when the PIR locates in the range of 1.3–3.5, individuals are more likely to have developing MetS. Also, among various marital status, the rate of MetS is highest in unmarried individuals. Additionally, the prevalence of MetS is higher in individuals who smoke, have a high BMI, experience short sleep duration, consume excessive calories, suffer from arthritis or heart disease, and engage in short durations of physical activity or inactive LTPA.

### Association between single LTPA or DQ with MetS and its componentshead

**Table 2** presents the effect of individual LTPA patterns and DQ on MetS after controlling for potential confounding factors. Compared with the inactive pattern, RA (AOR = 0.77, 95% CI: 0.69–0.85) was negatively associated with MetS. Moreover, RA negatively correlated with MetS components. Specifically, RA was significantly negatively associated with waist circumference (AOR = 0.70, 95% CI: 0.64–0.76), fasting glucose (AOR = 0.78, 95% CI: 0.67–0.89), blood pressure (AOR = 0.82, 95% CI: 0.73–0.92), triglyceride (AOR = 0.89, 95% CI: 0.81–0.98), and high-density lipoprotein cholesterol (AOR = 0.82, 95% CI: 0.75–0.90). However, no significant associations were observed between WW, IA, and MetS and its components (S1-S5 Tables in S1 File).

**Table 2:** Association of leisure time physical activity or diet quality with MetS, NHANES 2007-2018 (N = 31,482).

Variables	Model 1 (95% CI)	Model 2 (95% CI)	Model 3 (95% CI)
Leisure time physical activity pattern			
Inactive	Ref	Ref	Ref
Insufficiently active	0.79 (0.70,0.90)	0.86 (0.74,0.99)	0.91 (0.78,1.06)
Weekend warrior	0.68 (0.54,0.86)	0.76 (0.59,0.98)	0.88 (0.66,1.16)
Regularly active	0.47 (0.43,0.51)	0.60 (0.55,0.67)	0.77 (0.69,0.85)
Dietary quality			
Low dietary quality (HEI-2015 < 60)	Ref	Ref	Ref
High dietary quality (HEI-2015 ≥ 60)	1.03 (0.95, 1.11)	0.74 (0.67,0.81)	0.87 (0.79,0.96)

Model 1 Unadjusted model.

Model 2 Survey cycle, sex, age, race, education, PIR, marital status.

Model 3 Survey cycle, sex, age, race, education, PIR, marital status, BMI, alcohol use, smoking, sleep duration, sedentary duration, total energy intake, arthritis and heart attack.

A statistically significant negative association was observed between HDQ (AOR = 0.87, 95% CI: 0.79–0.96) and MetS compared to LDQ (Table 2). Moreover, an association between DQ and MetS and its components was noted. Among them, HDQ was significantly negatively associated with waist circumferences (AOR = 0.84, 95% CI: 0.73–0.91), fasting glucose (AOR = 0.92, 95% CI: 0.80–0.99), blood pressure (AOR = 0.88, 95% CI: 0.74–0.95), triglyceride (AOR = 0.87, 95% CI: 0.79–0.95), and high-density lipoprotein cholesterol (AOR = 0.81, 95% CI: 0.73–0.94) (S1-S5 Tables in S1 File).

### Joint association between LTPA&DQ with MetS and its components

Compared with inactive&LDQ, RA&HDQ was significantly negatively associated with MetS (AOR = 0.63, 95% CI: 0.55–0.73), and waist circumference (AOR = 0.58, 95% CI: 0.52–0.65), fasting glucose (AOR = 0.75, 95% CI: 0.63–0.90), blood pressure (AOR = 0.67, 95% CI: 0.58–0.77), triglyceride (AOR = 0.83, 95% CI: 0.73–0.94), and high-density lipoprotein cholesterol (AOR = 0.69, 95%CI: 0.60–0.79). Similarly, IA&HDQ was significantly negatively associated with waist circumference (AOR = 0.81, 95% CI: 0.66–0.94) and blood pressure (AOR = 0.81, 95% CI: 0.71–0.92). In addition, WW&HDQ was significantly negatively correlated with fasting glucose (AOR = 0.58, 95% CI: 0.39–0.66) and blood pressure (AOR = 0.72, 95% CI: 0.64–0.87) (Table 3, S6-S10 Tables in S1 File).

**Table 3:** Joint association between leisure time physical activity & dietary quality with MetS, NHANES 2007-2018 (N = 31,482).

LTPA pattern * Diet quality	Model 1 (95%CI)	Model 2 (95%CI)	Model 3 (95%CI)
Inactive * LDQ	Ref	Ref	Ref
Insufficiently active * LDQ	0.81 (0.69,0.94) ^a^	0.87 (0.72,1.05)	0.90 (0.73,1.11)
WW * LDQ	0.71 (0.54,0.93) ^a^	0.79 (0.60,1.05)	0.93 (0.68,1.27)
Regularly active * LDQ	0.49 (0.44,0.55) ^a^	0.65 (0.57,0.73) ^a^	0.88 (0.81,1.01)
Inactive * HDQ	1.10 (0.98,1.23)	0.81 (0.71,0.92) ^a^	0.94 (0.82,1.08)
Insufficiently active * HDQ	0.83 (0.70,0.99) ^a^	0.65 (0.53,0.79) ^a^	0.83 (0.68,1.02)
WW * HDQ	0.64 (0.42,0.97) ^a^	0.51 (0.32,0.83) ^a^	0.68 (0.42,1.10)
Regularly active * HDQ	0.47 (0.41,0.53) ^a^	0.42 (0.36,0.49) ^a^	0.63 (0.55,0.73) ^a^

^a^ = *P* < 0.05.

Model 1 Unadjusted model.

Model 2 Survey cycle, sex, age, race, education, PIR, marital status.

Model 3 Survey cycle, sex, age, race, education, PIR, marital status, BMI, alcohol use, smoking, sleep duration, sedentary duration, total energy intake, arthritis and heart attack.

### Subgroup analysis

To determine whether the relationships varied across different demographic characteristics, stratification and interaction analyses were performed. S11 Table in S1 File illustrated the association between lifestyle factors and MetS remained consistent across the subgroups based on sex, race, PIR, education, and marital status, with no interaction effects observed (S11 Table in S1 File).

### Non-linear relationship between LTPA&DQ with MetS and its components

RCS regression was used to examine the non-linear relationship between total LTPA and MetS in more detail. Total LTPA was obtained by summing MPA and VPA time. A significant non-linear negative correlation was observed between total LTPA and MetS (*P* for non-linearity < 0.001). Moreover, the reduction in MetS risk was even more significant with 500 min of exercise per week, and the decline in MetS gradually leveled off with more than 500 min of exercise. Similar non-linear trends were observed among fasting glucose (*P* for non-linearity = 0.004), blood pressure (*P* for non-linearity < 0.001), and triglyceride (*P* for non-linearity = 0.01) (S1 Fig in S1 File).

A significant nonlinear dose-response association between HEI-2015 scores and MetS risk (*P* for non-linearity < 0.001), with risk reduction becoming progressively evident at higher dietary quality scores. Similar nonlinear patterns were identified for specific MetS components: fasting glucose (*P* for non-linearity = 0.003), blood pressure (*P* for non-linearity = 0.002), triglyceride (P for non-linearity < 0.001), and high-density lipoprotein cholesterol (*P* for non-linearity < 0.001) (S2 Fig in S1 File).

## Discussion

This study examined the relationship between LTPA, DQ, and their combinations with MetS and its components. RA and HDQ were significantly negatively associated with MetS alone, indicating that both RA and HDQ are beneficial in reducing the risk of MetS. Compared with the inactive&LDQ, the RA&HDQ and IA&HDQ were significantly associated with a reduced risk of MetS. Although the WW&HDQ significantly reduced the risk of fasting glucose and blood pressure, it was not significantly associated with MetS overall. This suggested that although the WW&HDQ could be effective for specific components, it is not insufficient to substantially improve the overall risk of MetS when used alone.

### Participants characteristics

This study found that males might be more prone to developing adverse lifestyle habits compared to females, such as smoking and alcohol consumption, which are established factors associated with an increased prevalence of MetS [30]. Moreover, Mexican American exhibited a higher risk of developing MetS compared to other racial groups, suggesting that racial factors may play a significant role in the pathogenesis of MetS, which has been corroborated by previous research [31]. Also, individuals of lower socioeconomic status faced harder access to healthy diets and often chose high-calorie, high-fat, and high-sugar foods, which may be linked to MetS development [32]. Additionally, individuals who have never married may face a higher risk of developing MetS. This could be attributed to factors such as life stress, inadequate social support, and a lack of health-promoting behaviors among unmarried individuals [33]. Furthermore, smoking, a high BMI, inadequate sleep, excessive calorie intake, a history of arthritis or heart disease, and sedentary behavior or insufficient physical activity all could significantly elevate an individual’s risk of developing MetS [34].

### Association between single LTPA or DQ with MetS and its components

This study found that RA was negatively correlated with MetS and its components [35,36]. Compared with inactive, IA, and WW, RA—defined as engaging in LTPA three or more times per week—was more effective in reducing MetS risk. This finding is consistent with previous studies [12,37]. Compared to WW, persistent RA is associated with higher levels of high-density lipoprotein cholesterol [38,39], lower levels of triglycerides [40], and lower blood pressure. However, it is difficult to achieve the beneficial effects against MetS with less frequent exercise, which may be a potential explanation for why only RA was significantly negatively associated with MetS.

In addition, our findings revealed that HDQ was significantly negatively associated with MetS, consistent with previous studies [38]. The main predisposing factor for MetS is an unhealthy diet, characterized by a high intake of saturated fatty acids, trans fatty acid isomers, single carbohydrates, and salts [41], which may interfere with metabolic processes in the body. A 15-year study found that individuals with LDQ had a double risk of insulin resistance [42] and a significantly increased risk of MetS [43].

### Joint association between LTPA and DQ with MetS and its components

MetS is a complex condition involving multiple metabolic abnormalities. Improving MetS often requires more than a single intervention. Therefore, this study examined the effects of eight combinations of LTPA and DQ, and found that both RA&HDQ and IA&HDQ were significantly negatively associated with MetS and its components.

RA&HDQ was negatively associated with MetS and its components. A study has demonstrated that, in comparison to merely improving exercise patterns or modifying diet alone, the combined approach of utilizing RA and HDQ exhibits a stronger effect in reducing health risks [19]. RA &HDQ can control weight by increasing energy consumption and reducing intake of high-calorie, high-carbohydrate foods, thereby creating a “negative energy balance” that helps reduce the likelihood of central obesity in individuals with MetS [44]. At the molecular level, RA&HDQ can effectively regulate oxidative stress processes [45,46]. By increasing the activity of antioxidant enzymes in the body, RA can improve the ability of cells to remove free radicals, thereby reducing oxidative stress damage to DNA, proteins, and lipids [47]. HDQ directly neutralizes free radicals by supplying antioxidants, such as vitamins, minerals, and polyphenols, which prevent them from damaging cellular structure and function[48]. This dual approach highlights the synergistic effect of RA&HDQ in reducing oxidative stress. In addition, RA&HDQ can regulate inflammatory responses promoted by MetS through a complex network of molecules [49]. RA promotes the production of anti-inflammatory cytokines and inhibits the release of pro-inflammatory cytokines, which helps to maintain the balance of inflammatory responses [50]. Certain nutrients in HDQ, such as Omega-3 fatty acids and dietary fiber, can also reduce the damage of inflammation by affecting the synthesis and signaling of inflammatory mediators [51]. This shared regulatory mechanism underscores the common anti-inflammatory effects of exercise and a healthy diet.

IA&HDQ was significantly negatively associated with waist circumference, and high-density lipoprotein cholesterol levels. This outcome may be due to the more pronounced effect of a healthy diet compared to exercise. Adjusting dietary patterns—such as reducing the intake of high-calorie, high-fat, and high-sugar foods and increasing the in-take of nutritious foods such as vegetables, fruits, and whole grains—can lead to more rapid weight loss and improve fat distribution in the body [52]. In contrast, exercise primarily functions by enhancing energy expenditure, continually burning excess calories, and decreasing fat accumulation [53]. This may explain why IA&HDQ was significantly and negatively correlated with MetS, waist circumference, and high-density lipoprotein cholesterol levels.

Previous studies have shown that WW can reduce the risk of depression [54], lower abdominal obesity [12], and lower certain health risks [53]. However, in this study, WW was not significantly associated with MetS, although it achieved the recommended LTPA duration. There are several possible reasons for these differences. First, an earlier study reported that the number and frequency of LTPA are more important than the duration. Patients are advised to engage in LTPA multiple times per week rather than increasing the duration of a single session of exercise [55]. For many chronic diseases, the accumulation of exercise effects takes time and sustained effort, and WW may not be suitable for long-term regular exercise. Moreover, WW typically requires the body to endure a significant exercise load within a short period due to a general lack of regular exercise stimulation [54]. In addition, research has shown that individuals performing WW are significantly more likely to develop MetS compared to those participating in RA exercise [55], suggesting that WW may contribute to metabolic disorders. Moreover, one study found that either aerobic exercise or resistance exercise, carried out at least 40–45 min per session 3 times a week, could effectively improve hepatic steatosis [54]. WW twice weekly may not be adequate to lower the risk of metabolic syndrome. Although the integration of WW and HDQ failed to diminish the risk of metabolic syndrome, it did provide advantages in terms of reducing fasting blood glucose levels and mitigating blood pressure risks [56], regardless of the frequency of exercise. It is important to note that, although WW&HDQ can reduce blood pressure and blood sugar levels, its effect may not be as stable as that of long-term RA combined with HDQ.

### Strengths and limitations

This study has several innovative advantages. First, it is based on data from NHANES, which ensures a significant large sample size and the validity of the findings. Second, we constructed multiple weighted multivariate logistic regression models, adjusted for multiple variables, to robustly assess the relationship between LTPA, DQ, and MetS and its components. However, this study also has certain limitations. First, as a cross-sectional study, it cannot infer a causal relationship between LTPA, DQ, and MetS. Second, although we adjusted for various potential confounders, the possibility of unmeasured confounders could not be excluded.

This research not only elucidates the mechanism of health promotion but also provides a scientific basis for assessing MetS risk. Future studies should focus on exploring specific molecular pathways and interactions involved, with a view to making more breakthroughs in MetS prevention and treatment.

## Conclusion

The results of this study found that RA&HDQ is significantly negatively associated with MetS. Although WW&HDQ significantly reduces the risk of fasting glucose and blood pressure, no significant associations are observed with MetS, which suggests that it may not be sufficient for the overall benefit of improving MetS. RA&LDQ significantly reduces the risk of elevated waist circumference. The decline in MetS risk becomes even more significant with exercise for 500 min/week. Similar trends are observed among fasting glucose, blood pressure. and triglyceride.

Based on the findings, RA&HDQ is strongly recommended as a primary lifestyle guideline for MetS prevention, especially for high-risk populations. For patients with specific metabolic abnormalities, WW&HDQ may be considered to improve fasting glucose levels and blood pressure control. These findings may contribute to the development of personalized dietary and exercise guidelines for MetS prevention in both clinical and public health settings.

## Supporting information

S1 File Supplementary tables. (DOCX)

S2 File Raw data. (CSV)
