# Supplementary material for: Whether joint leisure time physical activity and dietary quality alleviates metabolic syndrome and its components: evidence from the National Health and Nutrition Examination Survey (2007–2018)
Source: PLoS One. 2025 May 6;20(5):e0322608. doi: 10.1371/journal.pone.0322608 (PMC12054862; doi:10.1371/journal.pone.0322608)
Supplement: S1 File — (DOCX) [file pone.0322608.s001.docx]

**Whether joint leisure time physical activity and dietary quality alleviates metabolic syndrome and its components: evidence from the National Health and Nutrition Examination Survey (2007–2018)**

**Supporting information**

**Contents**

**[1. Association between single LTPA or DQ with MetS components 2](#_Toc21886)**

**[2. Joint association between LTPA&DQ with MetS and its components 5](#_Toc2283)**

**[3. Subgroup analysis 8](#_Toc19317)**

**[4. Non-linear relationship between total MVPA or DQ and the risk of MetS 10](#_Toc18967)**

# 1. Association between single LTPA or DQ with MetS components

**S1 Table.** Association of leisure time physical activity or diet quality and waist circumference, NHANES 2007-2018 (N = 31,482)

| Variables | Model 1 (95%CI) | Model 2 (95%CI) | Model 3 (95%CI) |
| --- | --- | --- | --- |
| LTPA pattern |  |  |  |
| Inactive | Ref | Ref | Ref |
| Insufficiently active | 1.08 (0.95,1.24) | 1.02 (0.89,1.17) | 1.03 (0.90,1.18) |
| weekend warrior | 0.71 (0.58,0.86) | 0.82 (0.67,1.01) | 0.81 (0.66,1.01) |
| Regularly active | 0.55 (0.51,0.60) | 0.69 (0.63,0.75) | **0.70 (0.64,0.76)** |
| Diet quality |  |  |  |
| Low dietary quality (HEI-2015 < 60) | Ref | Ref | Ref |
| High dietary quality (HEI-2015≥ 60) | 0.65 (0.52,0.78) | 0.80 (0.73,0.86) | **0.84 (0.73,0.91)** |

Model 1 Unadjusted model

Model 2 Survey cycle, sex, age, race, education, PIR, marital status

Model 3 Survey cycle, sex, age, race, education, PIR, marital status, BMI, alcohol use, smoking, sleep duration, sedentary duration, total energy intake, arthritis and heart attack.

**S2 Table.** Association of leisure time physical activity or diet quality and fasting glucose , NHANES 2007-2018 (N = 31,482)

| Variables | Model 1 (95%CI) | Model 2 (95%CI) | Model 3 (95%CI) |
| --- | --- | --- | --- |
| LTPA pattern |  |  |  |
| Inactive | Ref | Ref | Ref |
| Insufficiently active | 0.74 (0.64,0.86) | 0.87 (0.75,1.02) | 0.94 (0.80,1.10) |
| weekend warrior | 0.51 (0.37,0.69) | 0.68 (0.48,0.97) | 0.79 (0.56,1.11) |
| Regularly active | 0.42 (0.37,0.49) | 0.63 (0.55,1.73) | **0.78 (0.67,0.89)** |
| Diet quality |  |  |  |
| Low dietary quality (HEI-2015 < 60) | Ref | Ref | Ref |
| High dietary quality (HEI-2015≥ 60) | 0.67 (0.47,0.79) | 0.85 (0.75,0.97) | **0.92 (0.80, 0.99)** |

Model 1 Unadjusted model

Model 2 Survey cycle, sex, age, race, education, PIR, marital status

Model 3 Survey cycle, sex, age, race, education, PIR, marital status, BMI, alcohol use, smoking, sleep duration, sedentary duration, total energy intake, arthritis and heart attack.

**S3 Table.** Association of leisure time physical activity or diet quality and blood pressure, NHANES 2007-2018 (N = 31,482)

| Variables | Model 1 (95%CI) | Model 2 (95%CI) | Model 3 (95%CI) |
| --- | --- | --- | --- |
| LTPA pattern |  |  |  |
| Inactive | Ref | Ref | Ref |
| Insufficiently active | 0.76 (0.67,0.87) | 0.81 (0.70,0.94) | 0.85 (0.73,1.03) |
| weekend warrior | 0.54 (0.46,0.64) | 0.72 (0.58,0.89) | 0.81 (0.65,1.01) |
| Regularly active | 0.46 (0.43,0.51) | 0.68 (0.61,0.76) | **0.82 (0.73,0.92)** |
| Diet quality |  |  |  |
| Low dietary quality (HEI-2015 < 60) | Ref | Ref | Ref |
| High dietary quality (HEI-2015≥ 60) | 0.53 (0.39,0.81) | 0.76 (0.68, 0.84) | **0.88 (0.74,0.95)** |

Model 1 Unadjusted model

Model 2 Survey cycle, sex, age, race, education, PIR, marital status

Model 3 Survey cycle, sex, age, race, education, PIR, marital status, BMI, alcohol use, smoking, sleep duration, sedentary duration, total energy intake, arthritis and heart attack.

**S4 Table.** Association of leisure time physical activity or diet quality and triglycerides, NHANES 2007-2018 (N = 31,482)

| Variables | Model 1 (95%CI) | Model 2 (95%CI) | Model 3 (95%CI) |
| --- | --- | --- | --- |
| LTPA pattern |  |  |  |
| Inactive | Ref | Ref | Ref |
| Insufficiently active | 1.04 (0.91,1.17) | 1.05 (0.92,1.20) | 1.08 (0.95,1.24) |
| weekend warrior | 0.85 (0.69,1.04) | 0.85 (0.69,1.04) | 0.91 (0.73,1.13) |
| Regularly active | 0.69 (0.64,0.75) | 0.78 (0.71,0.86) | **0.89 (0.81,0.98)** |
| Diet quality |  |  |  |
| Low dietary quality (HEI-2015 < 60) | Ref | Ref | Ref |
| High dietary quality (HEI-2015≥ 60) | 0.74 (0.50,0.81) | 0.86 (0.79,0.93) | **0.87 (0.79, 0.95)** |

Model 1 Unadjusted model

Model 2 Survey cycle, sex, age, race, education, PIR, marital status

Model 3 Survey cycle, sex, age, race, education, PIR, marital status, BMI, alcohol use, smoking, sleep duration, sedentary duration, total energy intake, arthritis and heart attack.

**S5 Table.** Association of leisure time physical activity or diet quality and high-density lipoprotein cholesterol, NHANES 2007-2018 (N = 31,482)

| Variables | Model 1 (95%CI) | Model 2 (95%CI) | Model 3 (95%CI) |
| --- | --- | --- | --- |
| LTPA pattern |  |  |  |
| Inactive | Ref | Ref | Ref |
| Insufficiently active | 0.86 (0.73,1.02) | 0.90 (0.76,1.06) | 0.92 (0.77,1.09) |
| weekend warrior | 1.06 (0.83,1.34) | 0.83 (0.65,1.06) | 0.90 (0.70,1.15) |
| Regularly active | 0.76 (0.68,0.84) | 0.72 (0.65,0.79) | **0.82 (0.75,0.90)** |
| Diet quality |  |  |  |
| Low dietary quality (HEI-2015 < 60) | Ref | Ref | Ref |
| High dietary quality (HEI-2015≥ 60) | 0.69 (0.63,0.75) | 0.76 (0.69,0.84) | **0.81 (0.73,0.94)** |

Model 1 Unadjusted model

Model 2 Survey cycle, sex, age, race, education, PIR, marital status

Model 3 Survey cycle, sex, age, race, education, PIR, marital status, BMI, alcohol use, smoking, sleep duration, sedentary duration, total energy intake, arthritis and heart attack.

# **2. Joint association between LTPA&DQ with MetS and its components**

**S6 Table.** Joint Association between LTPQ&DQ with waist circumstance, NHANES 2007-2018 (N = 31,482)

| LTPA pattern * Diet quality | Model 1 (95%CI) | Model 2 (95%CI) | Model 3 (95%CI) |
| --- | --- | --- | --- |
| Inactive * Low dietary quality | Ref | Ref | Ref |
| Insufficiently active * Low dietary quality | 1.19 (1.02,1.40) ^a^ | 1.18 (1.02,1.37) ^a^ | 1.18 (1.02,1.38) ^a^ |
| weekend warrior * Low dietary quality | 0.70 (0.55,0.89) ^a^ | 0.79 (0.62,1.01) | 0.78 (0.61,1.01) |
| Regularly active * Low dietary quality | 0.55 (0.47,0.63) ^a^ | 0.74 (0.64,0.86) ^a^ | 0.89 (0.71,1.01) ^a^ |
| Inactive * High dietary quality | 1.26 (1.14,1.40) ^a^ | 0.89 (0.79,0.99) ^a^ | 0.91 (0.81,1.02) |
| Insufficiently active * High dietary quality | 1.23 (1.02,1.48) ^a^ | 0.77 (0.62,0.96) ^a^ | **0.81 (0.66,0.94) ^a^** |
| weekend warrior * High dietary quality | 0.89 (0.68,1.19) | 0.77 (0.56,1.07) | 0.78 (0.56,1.09) |
| Regularly active * High dietary quality | 0.70 (0.63,0.78) ^a^ | 0.55 (0.49,0.62) ^a^ | **0.58 (0.52,0.65) ^a^** |

Note: ^a^=*P*<0.05

Model 1 Unadjusted model

Model 2 Survey cycle, sex, age, race, education, PIR, marital status

Model 3 Survey cycle, sex, age, race, education, PIR, marital status, BMI, alcohol use, smoking, sleep duration, sedentary duration, total energy intake, arthritis and heart attack.

**S7 Table.** Joint Association between LTPA &DQ with fast glucose, NHANES 2007-2018 (N = 31,482)

| LTPA pattern * Diet quality | Model 1 (95%CI) | Model 2 (95%CI) | Model 3 (95%CI) |
| --- | --- | --- | --- |
| Inactive * Low dietary quality | Ref | Ref | Ref |
| Insufficiently active * Low dietary quality | 0.82 (0.65,1.05) | 1.00 (0.78,1.29) | 1.04 (0.80,1.34) |
| weekend warrior * Low dietary quality | 0.64 (0.43,0.96) ^a^ | 0.92 (0.58,1.46) | 1.09 (0.68,1.74) |
| Regularly active * Low dietary quality | 0.44 (0.36,0.54) ^a^ | 0.69 (0.56,0.86) ^a^ | 0.85 (0.68,1.04) |
| Inactive * High dietary quality | 1.31 (1.15,1.48) | 0.95 (0.82,1.09) | 1.04 90.90,1.21) |
| Insufficiently active * High dietary quality | 0.88 (0.74,1.06) | 0.73 (0.59,0.90) ^a^ | 0.89 (0.72,1.10) |
| weekend warrior * High dietary quality | 0.48 (0.32,0.72) ^a^ | 0.44 (0.29,0.68) ^a^ | **0.58 (0.39,0.66) ^a^** |
| Regularly active * High dietary quality | 0.54 (0.45,0.64) ^a^ | 0.55 (0.46,0.67) ^a^ | **0.75 (0.63,0.90) ^a^** |

Note: ^a^=*P*<0.05

Model 1 Unadjusted model

Model 2 Survey cycle, sex, age, race, education, PIR, marital status

Model 3 Survey cycle, sex, age, race, education, PIR, marital status, BMI, alcohol use, smoking, sleep duration, sedentary duration, total energy intake, arthritis and heart attack.

**S8 Table.** Joint Association LTPA&DQ and blood pressure, NHANES 2007-2018 (N = 31,482)

| LTPA pattern * Diet quality | Model 1 (95%CI) | Model 2 (95%CI) | Model 3 (95%CI) |
| --- | --- | --- | --- |
| Inactive * Low dietary quality | Ref | Ref | Ref |
| Insufficiently active * Low dietary quality | 0.77 (0.64,0.94) ^a^ | 0.85 (0.65,1.10) | 0.87 (0.67,1.13) |
| weekend warrior * Low dietary quality | 0.57 (0.44,0.73) ^a^ | 0.78 (0.58,1.06) | 0.89 90.65,1.20) |
| Regularly active * Low dietary quality | 0.50 (0.44,0.58) ^a^ | 0.86 (0.75,0.98) ^a^ | 1.02 (0.88,1.18) |
| Inactive * High dietary quality | 1.41 (1.28,1.56) | 0.88 (0.78,1.01) | 0.98 (0.86,1.11) |
| Insufficiently active * High dietary quality | 1.05 (0.90,1.23) | 0.67 (0.56,0.80) ^a^ | **0.81 (0.71,0.92) ^a^** |
| weekend warrior * High dietary quality | 0.72 (0.57,0.91) ^a^ | 0.56 (0.42,0.76) ^a^ | **0.72 (0.64,0.87) ^a^** |
| Regularly active * High dietary quality | 0.61 (0.55,0.69) ^a^ | 0.50 (0.44,0.57) ^a^ | **0.67 (0.58,0.77) ^a^** |

Note: ^a^=*P*<0.05

Model 1 Unadjusted model

Model 2 Survey cycle, sex, age, race, education, PIR, marital status

Model 3 Survey cycle, sex, age, race, education, PIR, marital status, BMI, alcohol use, smoking, sleep duration, sedentary duration, total energy intake, arthritis and heart attack.

**S9 Table.** Joint Association between LTPA&DQ with triglyceride, NHANES 2007-2018 (N = 31,482)

| LTPA pattern * Diet quality | Model 1 (95%CI) | Model 2 (95%CI) | Model 3 (95%CI) |
| --- | --- | --- | --- |
| Inactive * Low dietary quality | Ref | Ref | Ref |
| Insufficiently active * Low dietary quality | 1.08 (0.90,1.27) | 1.09 (0.91,1.30) | 1.09 (0.90,1.33) |
| weekend warrior * Low dietary quality | 0.86 (0.64,1.16) | 0.85 (0.64,1.14) | 0.89 (0.66,1.22) |
| Regularly active * Low dietary quality | 0.72 (0.63,0.83) | 0.84 (0.73,0.97) ^a^ | 0.95 (0.82,1.11) |
| Inactive * High dietary quality | 1.07 (0.96,1.19) | 0.91 (0.81,1.01) | 0.99 (0.88,1.11) |
| Insufficiently active * High dietary quality | 1.06 (0.91,1.24) | 0.91 (0.77,1.08) | 1.05 (0.88,1.25) |
| weekend warrior * High dietary quality | 0.89 (0.67,1.18) | 0.76 (0.57,1.03) | 0.91 (0.67,1.23) |
| Regularly active * High dietary quality | 0.71 (0.64,0.79) ^a^ | 0.66 (0.58,0.75) ^a^ | **0.83 (0.73,0.94) ^a^** |

Note: ^a^=*P*<0.05

Model 1 Unadjusted model

Model 2 Survey cycle, sex, age, race, education, PIR, marital status

Model 3 Survey cycle, sex, age, race, education, PIR, marital status, BMI, alcohol use, smoking, sleep duration, sedentary duration, total energy intake, arthritis and heart attack.

**S10 Table.** Joint Association between LTPA&DQ with high-density lipoprotein cholesterol , NHANES 2007-2018 (N = 31,482)

| LTPA pattern * Diet quality | Model 1 (95%CI) | Model 2 (95%CI) | Model 3 (95%CI) |
| --- | --- | --- | --- |
| Inactive * Low dietary quality | Ref | Ref | Ref |
| Insufficiently active * Low dietary quality | 0.93 (0.77,1.13) | 0.95 (0.78,1.16) | 0.94 (0.76,1.16) |
| weekend warrior * Low dietary quality | 1.15 (0.85,1.56) | 0.91 (0.67,1.23) | 0.97 (0.70,1.33) |
| Regularly active * Low dietary quality | 0.81 (0.70,0.94) ^a^ | 0.74 (0.62,0.87) ^a^ | 0.84 (0.72,1.00) |
| Inactive * High dietary quality | 0.75 (0.66,0.85) ^a^ | 0.80 (0.71,0.91) ^a^ | 0.87 (0.76,1.01) |
| Insufficiently active * High dietary quality | 0.58 (0.47,0.73) ^a^ | 0.67 (0.53,0.84) ^a^ | 0.77 (0.61,1.96) |
| weekend warrior * High dietary quality | 0.68 (0.47,0.99) ^a^ | 0.58 (0.39,0.86) ^a^ | 0.69 (0.46,1.03) |
| Regularly active * High dietary quality | 0.52 (0.46,0.60) ^a^ | 0.55 (0.48,0.63) ^a^ | **0.69 (0.60,0.79) ^a^** |

Note: ^a^=*P*<0.05

Model 1 Unadjusted model

Model 2 Survey cycle, sex, age, race, education, PIR, marital status

Model 3 Survey cycle, sex, age, race, education, PIR, marital status, BMI, alcohol use, smoking, sleep duration, sedentary duration, total energy intake, arthritis and heart attack.

# **Subgroup analysis**

**S11 Table.** Subgroup analysis of the association between LTPA, DQ with MetS

| Subgroups | Inactive * Low dietary quality | Insufficiently active * Low dietary quality | weekend warrior * Low dietary quality | Regularly active * Low dietary quality | Inactive * High dietary quality | Insufficiently active * High dietary quality | weekend warrior * High dietary quality | Regularly active * High dietary quality | P for interaction |
| --- | --- | --- | --- | --- | --- | --- | --- | --- | --- |
| Sex |  |  |  |  |  |  |  |  | 0.500 |
| Men | Ref | 0.90 (0.66,1.23) | 0.96 (0.61,1.50) | 0.81 (0.65,1.02) | 0.89 (0.75,1.06) | 0.79 (0.59,1.07) | 0.63 (0.44,0.89) | 0.60 (0.50,0.72) |  |
| Women | Ref | 1.09 (0.77,1.54) | 0.67 (0.35,1.30) | 1.06 (0.82,1.38) | 1.01 (0.88,1.16) | 0.82 (0.66,1.03) | 1.04 (0.46,2.38) | 0.65 (0.52,0.81) |  |
| Race |  |  |  |  |  |  |  |  | 0.234 |
| Non-Hispanic White | Ref | 0.95 (0.68,1.32) | 0.94 (0.55,1.61) | 0.90 (0.71,1.12) | 0.86 (0.75,0.98) | 0.73 (0.58,0.92) | 0.65 (0.38,1.12) | 0.58 (0.48,0.69) |  |
| Non-Hispanic Black | Ref | 1.11 (0.76,1.64) | 1.19 (0.66,2.13) | 1.00 (0.73,1.38) | 1.56 (1.25,1.95) | 1.26 (0.89,1.79) | 1.23 (0.42,3.64) | 0.95 (0.73,1.23) |  |
| Mexican American | Ref | 1.03 (0.70,1.53) | 1.93 (0.97,3.84) | 0.97 (0.67,1.40) | 0.94 (0.72,1.23) | 0.42 (0.25,0.70) | 0.88 (0.51,1.53) | 0.82 (0.57,1.19) |  |
| Other Hispanic | Ref | 0.80 (0.39,1.64) | 0.52 (0.19,1.40) | 1.27 (0.74,2.17) | 1.02 (0.69,1.51) | 1.27 (0.77,2.11) | 0.59 (0.25,1.39) | 0.58 (0.34,0.98) |  |
| Other races | Ref | 0.95 (0.55,1.64) | 0.87 (0.32,2.31) | 0.98 (0.50,1.92) | 1.05 (0.61,1.80) | 1.08 (0.62,1.87) | 0.67 (0.27,1.66) | 0.69 (0.42,1.15) |  |
| Education level |  |  |  |  |  |  |  |  | .0.269 |
| < High school | Ref | 1.06 (0.66,1.70) | 1.25 (0.67,2.32) | 1.22 (0.92,1.61) | 0.90 (0.76,1.08) | 0.80 (0.51,1.26) | 1.37 (0.48,3.91) | 0.90 (0.68,1.20) |  |
| High school | Ref | 0.81 (0.53,1.26) | 1.02 (0.58,1.79) | 0.77 (0.54,1.10) | 1.10 (0.83,1.45) | 0.88 (0.61,1.27) | 0.53 (0.26,1.10) | 0.76 90.56,1.03) |  |
| > High school | Ref | 0.97 (0.70,1.34) | 0.88 (0.52,1.50) | 0.88 (0.71,1.08) | 0.92 (0.79,1.06) | 0.75 (0.60,0.95) | 0.67 (0.44,1.04) | 0.56 (0.48,0.66) |  |
| PIR |  |  |  |  |  |  |  |  | 0.244 |
| <1.3 | Ref | 1.15 (0.74,1.81) | 0.91 (0.54,1.52) | 1.02 (0.79,1.33) | 1.09 (0.81,1.30) | 0.62 (0.42,0.90) | 0.93 (0.55,1.59) | 0.73 (0.56,0.96) |  |
| 1.3-3.5 | Ref | 0.94 (0.69,1.30) | 1.42 (0.91,2.21) | 0.89 (0.68,1.16) | 1.05 (0.86,1.28) | 0.94 (0.70,1.27) | 0.87 (0.43,1.76) | 0.73 (0.57,0.92) |  |
| >3.5 | Ref | 0.87 (0.54,1.39) | 0.70 (0.32,1.57) | 0.85 (0.61,1.18) | 0.78 (0.63,0.98) | 0.71 (0.51,0.99) | 0.55 (0.32,0.95) | 0.53 (0.42,0.68) |  |
| Marital status |  |  |  |  |  |  |  |  | 0.296 |
| Married or living with partner | Ref | 0.95 (0.70,1.29) | 1.02 (0.63,1.65) | 0.88 (0.69,1.11) | 0.92 (0.80,1.05) | 0.73 (0.57,0.93) | 0.65 (0.42,0.99) | 0.58 (0.49,0.67) |  |
| Widowed, divorced or separated | Ref | 1.28 (0.74,2.20) | 1.15 (0.56,2.36) | 1.06 (0.75,1.51) | 0.95 (0.71,1.26) | 1.30 (0.82,2.06) | 1.05 (0.34,3.20) | 0.83 (0.59,1.18) |  |
| Never married | Ref | 1.79 (0.52,1.19) | 0.71 (0.38,1.32) | 1.14 (0.81,1.61) | 1.10 (0.86,1.42) | 0.79 (0.55,1.12) | 0.82 (0.40,1.71) | 0.76 (0.55,1.07) |  |

# **Non-linear relationship between total MVPA and the risk of MetS**

**
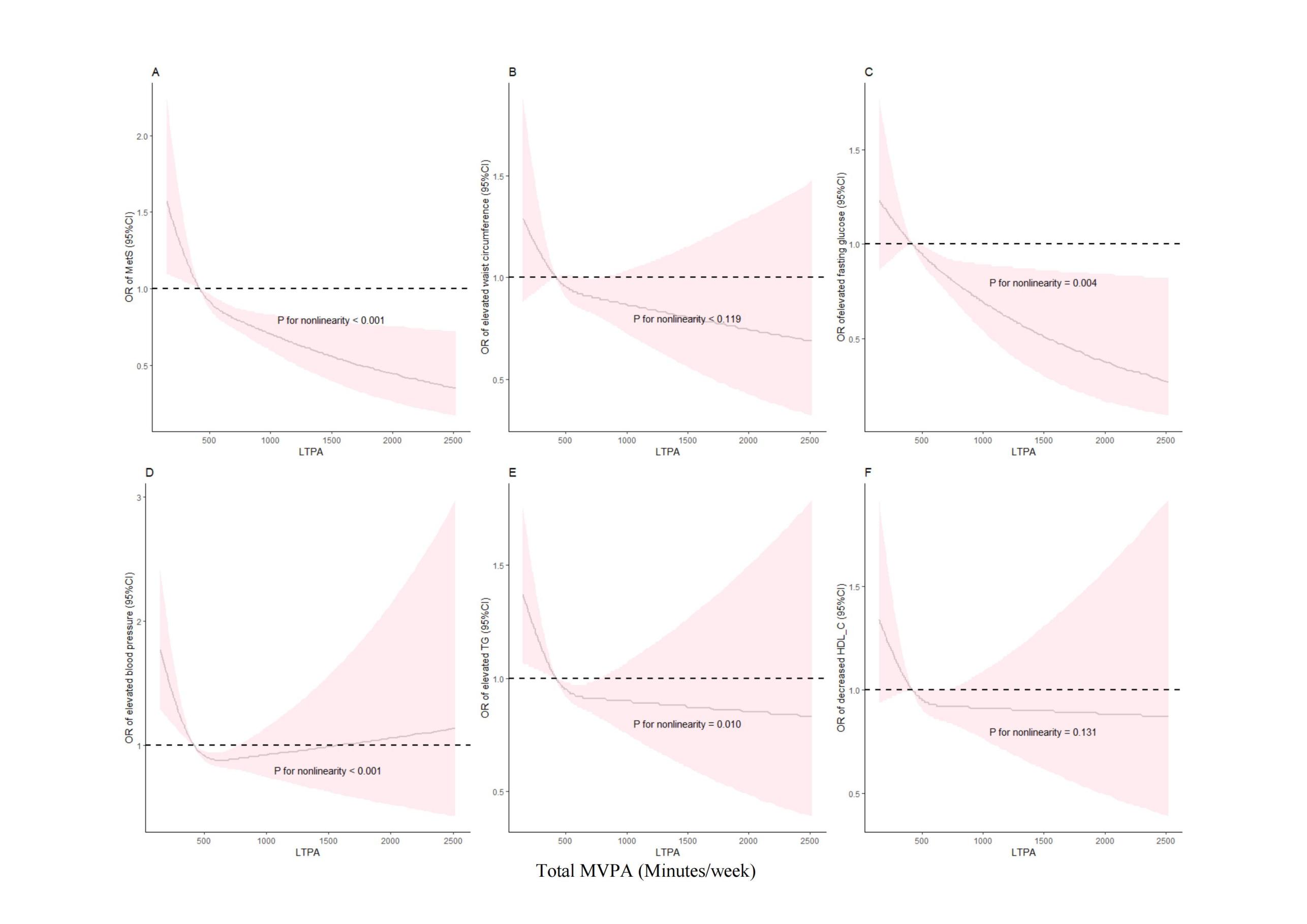
**

**S1 Fig.** Restricted cubic spline and 95%CI between total MVPA and the risk of metabolic syndrome, NHANES 2007–2018 (N = 31,482)


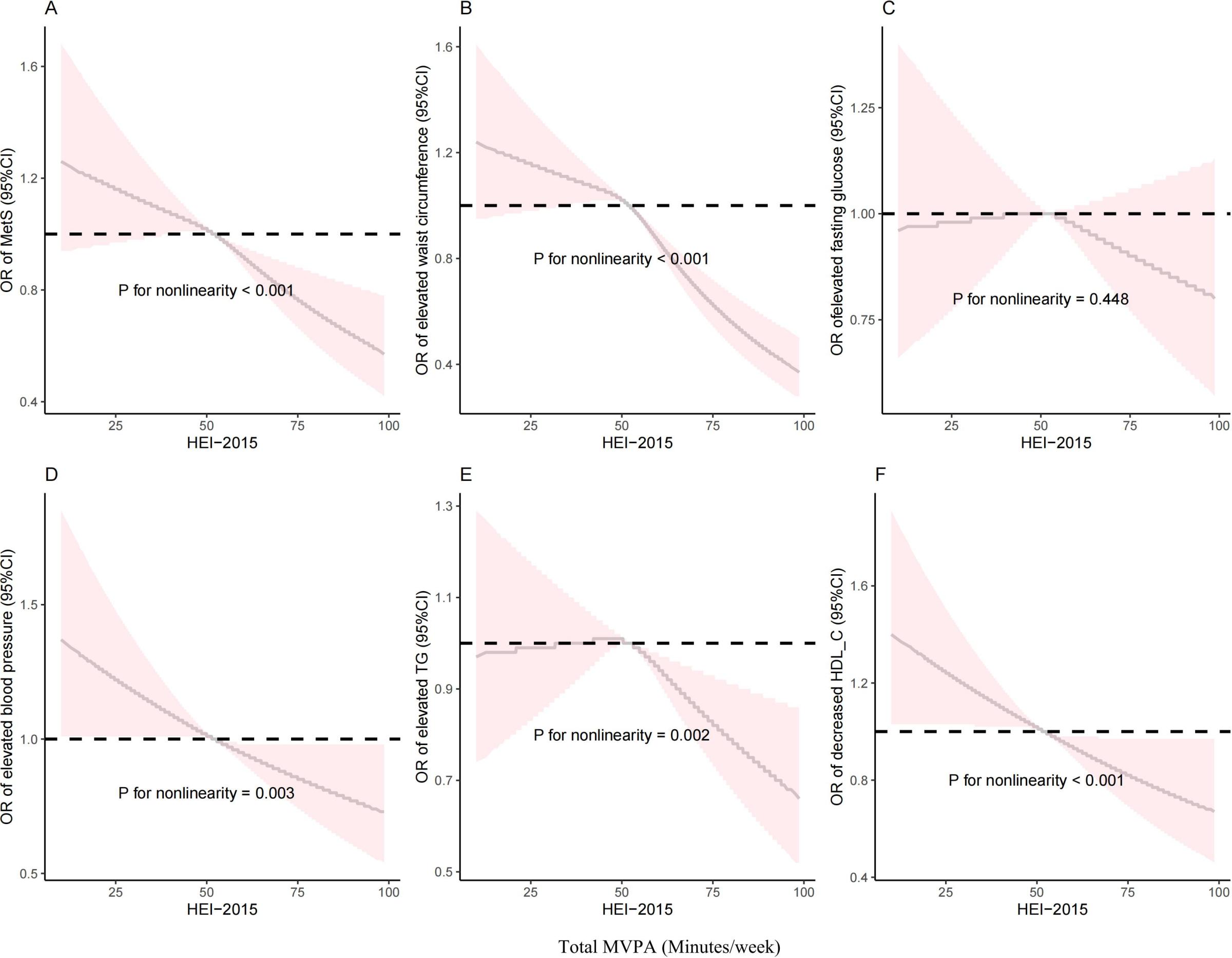


**S2 Fig.** Restricted cubic spline and 95%CI between total MVPA and the risk of metabolic syndrome, NHANES 2007–2018 (N = 31,482)
